# The implications of the American Board of Radiology's decision to relinquish its specialty board designation on prospective authorized medical physicists (AMPs) and radiation safety officers (RSOs)

**DOI:** 10.1002/acm2.70001

**Published:** 2025-02-21

**Authors:** Christopher J. Tien, Samantha J. Simiele, Joann I. Prisciandaro, Jacqueline E. Zoberi, Y. Jessica Huang, William A. Hinchcliffe, Hania A. Al‐Hallaq

**Affiliations:** ^1^ Department of Therapeutic Radiology Yale University School of Medicine New Haven Connecticut USA; ^2^ Department of Radiation Oncology Yale New Haven Hospital New Haven Connecticut USA; ^3^ Department of Radiation Oncology University of Alabama at Birmingham Birmingham Alabama USA; ^4^ Department of Radiation Oncology University of Michigan Ann Arbor Michigan USA; ^5^ Department of Radiation Oncology Washington University School of Medicine Saint Louis Missouri USA; ^6^ Department of Radiation Oncology Huntsman Cancer Hospital University of Utah Salt Lake City Utah USA; ^7^ Department of Radiation Safety Yale New Haven Hospital New Haven Connecticut USA; ^8^ Department of Radiation Oncology Emory University School of Medicine Atlanta Georgia USA

**Keywords:** american Board of Radiology, authorized medical physicist, diagnostic medical physics residency, medical physicis education, nuclear regulatory commission, radiation safety officer, residency, specialty board, therapeutic medical physics residency

## Abstract

In order to independently supervise the medical use of byproduct material, physicists in the United States (US) must legally meet the qualifications defined by the Nuclear Regulatory Commission (NRC) in the 35th part of the tenth title of the Code of Federal Regulations (§ 10 CFR Part 35). The American Board of Radiology (ABR) relinquished its NRC‐recognized specialty board (NSB) status at the end of 2023, which eliminated the NSB application pathway for those who earn ABR certification in 2024 and beyond. While these changes in NSB status are not retroactive and will not affect eligibility for diplomates who already possess certificates, these changes will nonetheless have repercussions for those individuals who regularly provide training and experience (T&E) attestations to the NRC, such as residency program directors, brachytherapy rotation preceptors, or radiation safety officers. This article will focus on the repercussions for new authorized medical physicist and radiation safety officer applicants with ABR certificates to be conferred in 2024 and later.

## 1 BACKGROUND

In order to independently supervise the medical use of byproduct material, physicists in the United States (US) must legally meet the qualifications defined by the Nuclear Regulatory Commission (NRC) in the 35th part of the tenth title of the Code of Federal Regulations (§ 10 CFR Part 35).^1^ Medical use is defined by the NRC as the internal or external administration of byproduct material or the radiation from byproduct material to patients or human research subjects under the supervision of an authorized user.^2^ The NRC has delegated oversight to many States which have established regulations which meet or exceed § 10 CFR Part 35 requirements, known as Agreement States.^2^ In this article, discussions will refer to NRC regulations rather than State regulations.

**FIGURE 1:**
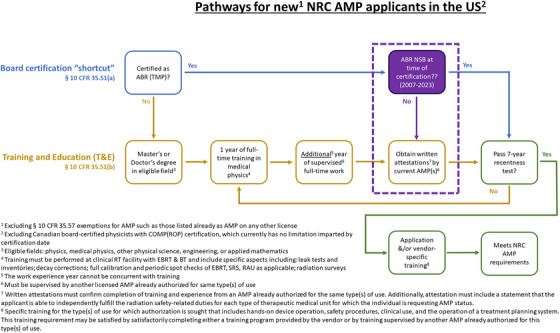
Pathways for new NRC AMP applicants in the US. BT, brachytherapy; EBRT, external beam radiotherapy; RAU, remote afterloader unit; SRS, stereotactic radiosurgery; TMP, Therapeutic medical physics.

**FIGURE 2:**
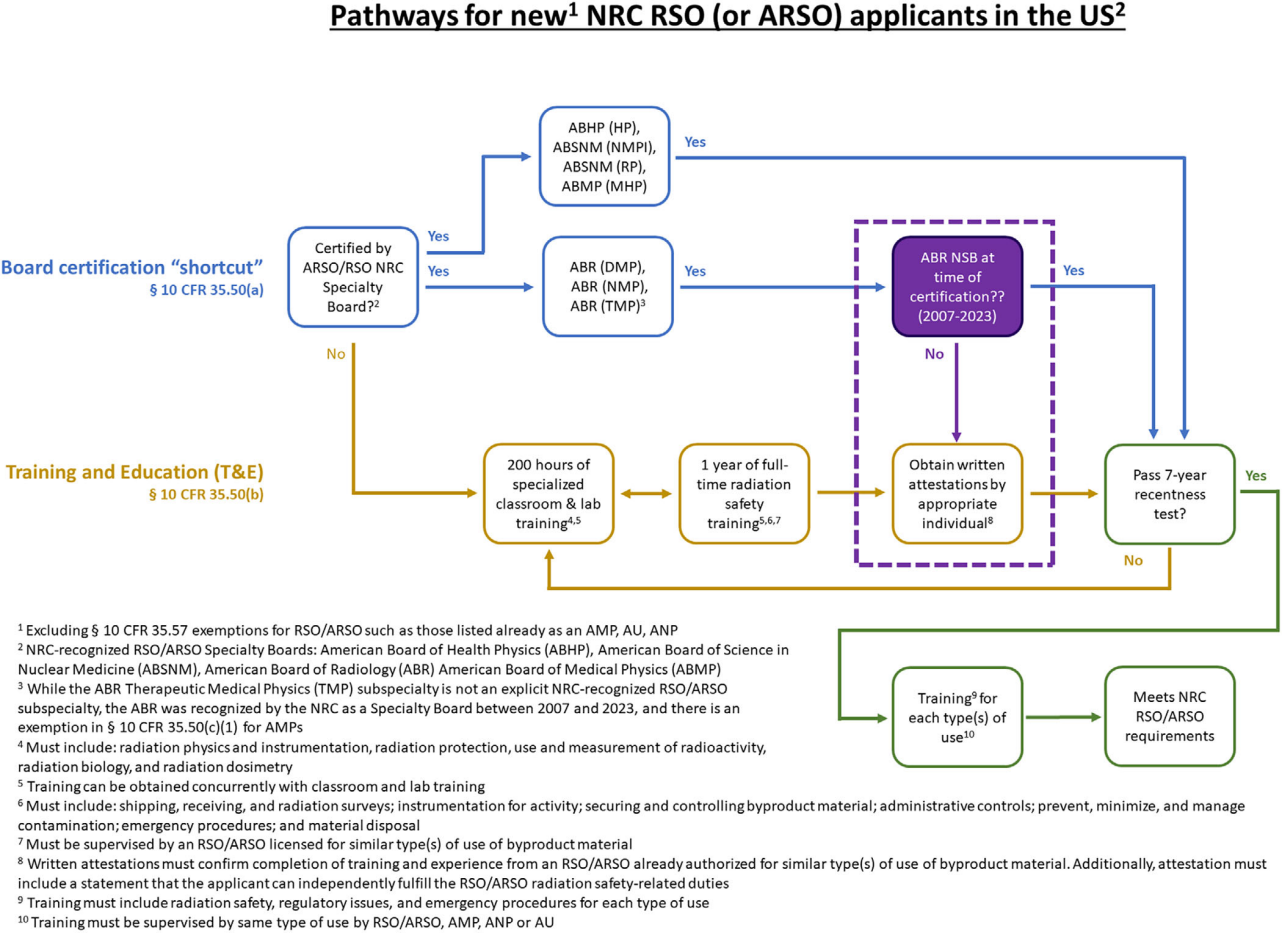
Pathways for new NRC RSO (or ARSO) applicants in the US.

Applications are evaluated based on a rubric of didactic education, clinical training, and experience for prospective AMPs (§ 10 CFR 35.51)^3^ and RSOs (§ 10 CFR 35.50).^4^ The application process is illustrated in Figures 1 and 2, and is overseen either directly by the federal NRC organization or by State agencies for NRC Agreement States. There is a prevailing “7‐year recency” clause,^5^ which only considers education and training if performed within 7 years of the application—however, this window can be lengthened with related continuing education and/or experience.

For physicists, the two relevant roles defined by the NRC are: (1) authorized medical physicist (AMP), who is an individual listed on an NRC or agreement state license or broad scope permit and is responsible for certain activities for: Remote afterloader unit (RAU) brachytherapy, Sr‐90 (for ophthalmic use), Teletherapy, and/or Gamma stereotactic radiosurgery use^3^ and (2) radiation safety officer (RSO)—or associate radiation safety officer (ARSO)—who is an individual responsible for implementation and management of the radiation protection program in accordance with 10 CFR 35.24.^4^ This article will focus on the RAU brachytherapy use for the AMP role, which is the only training experience required during residency. For the verbiage in this article, “RSO” will include the ARSO title.

Historically, regulations were established such that all applicants were evaluated based on the training and experience (T&E) route. Figures 1 and 2 illustrate the different evaluation processes exclusive to NSB diplomates including the new changes in the Board‐certification route.^3^, ^4^, ^5^ In order to ease the practical burden of inspecting an individual's T&E, in 2005, the NRC formally began to accept board certifications in lieu of documented T&E from a selected handful of NRC‐recognized Specialty Boards (NSBs) as summarized in Table 1.^6^ For physicists based in the US, there was only eligibility for AMP status through the ABR (Therapeutic Medical Physics subspecialty).^6^ Nominally, the Canadian College of Physicists in Medicine (CCPM) remains an alternative but is restricted to the subset of Canadian physicists practicing in the United States.^6^ On the other hand, there are still multiple other subspecialties which hold NSB status for RSO eligibility including the American Board of Health Physics, the American Board of Science in Nuclear Medicine, and the American Board of Medical Physics.^6^

**TABLE 1:** Nuclear regulatory commission's recognized specialty and subspecialty boards for authorized medical physicist (AMP) and radiation safety officer (RSO) applicants.

Role	Specialty Board	Subspecialty	Start date	End date
AMP	American Board of Radiology (ABR)	Therapeutic medical physics (TMP) aka therapeutic radiologic physics (TRP)	2007	2023
AMP	Canadian College of Physicists in Medicine (CCPM)	Radiation oncology physics (ROP)	2009	–
RSO	ABR	Diagnostic medical physics (DMP) aka diagnostic radiologic physics (DRP)	2007	2023
RSO	ABR	Nuclear medical physics (NMP) aka medical nuclear physics (MNP)	2007	2023
RSO	American Board of Health Physics (ABHP)	Health physics (HP)	2005	–
RSO	American Board of Science in Nuclear Medicine (ABSNM)	Nuclear medicine physics and instrumentation (NMPI)	2006	–
RSO	ABSNM	Radiation protection (RP)	2006	–
RSO	American Board of Medical Physics (ABMP)	Medical health physics (MHP)	2010	–

The American Board of Radiology (ABR) relinquished its NSB status at the end of 2023, which eliminated the NSB application pathway for those who earned ABR certification in 2024 and beyond.^6^ This article will focus on the repercussions for new AMP/RSO applicants with ABR certificates to be conferred in 2024 and later. While these changes in NSB status are not retroactive and will not affect eligibility for diplomates who already possess certificates—which contain the verbiage of “AMP eligible” or “RSO eligible”—these changes will nonetheless have repercussions for those individuals who regularly provide T&E attestations to the NRC, such as residency program directors, brachytherapy rotation preceptors, or radiation safety officers.^7^

There is a lack of consensus on AMP T&E requirements amongst program directors and employers for RAU brachytherapy use, with a healthy proportion of advocates for each side. In 2021, Aima et al. conducted a formal survey on physicist brachytherapy training of 110 program directors (PDs) of Commission on Accreditation of Medical Physics Educational Programs (CAMPEP) accredited therapeutic medical physics residency programs.^8^ Of the 55 PDs who responded, there was a fairly even split (57% yes vs. 43% no) amongst programs who attest to AMP T&E for their graduating residents,^8^ despite all CAMPEP‐accredited residencies adhering to the same minimum curricular standards. In fact, Aima et al. reported that 85% of residency PDs believed they would benefit from more structured guidelines for the AMP attestation process for RAU brachytherapy use.^8^ This survey was conducted in 2021, prior to the changes in the ABR NSB status in 2024. This article will recount a few of the differences in interpretation amongst programs.

## 2 DEFINING “TRAINING” AND “WORK EXPERIENCE”

With the withdrawal of the ABR as an NSB, all applicants—including those previously eligible for the board‐certified route through the ABR—can only become authorized through the T&E route. According to regulations within the T&E route, AMP applicants require 2 years of full‐time training and/or supervised experience in medical physics.^3^, ^4^ The AMP application is further regimented in that the time must be categorized as either training or work experience, with 1 year of each required to fulfill the T&E requirement.^3^ Notably, the work experience is not limited to time solely spent on the specific NRC use (i.e. RAU brachytherapy, ophthalmic Sr‐90, Teletherapy, and Gamma stereotactic radiosurgery use), as long as the supervisor meets the requirements of an AMP.^3^ Currently, ambiguity exists regarding whether full‐time experience during residency counts toward work experience.^3^ This nuanced difference between training and work experience is not relevant to RSO applicants, which broadly requires full‐time supervised “experience.^4^”

One potential agency to provide additional guidance on training versus experience is CAMPEP, as it is the primary accrediting body that defines the minimum standards for medical physics education and training.^9^ CAMPEP lends eponymous support for residencies to be classified as entirely training experience. Within its latest accreditation standards (revised July 2024), CAMPEP refers to residencies as clinical training (Sections 2.2 and 2.14)^9^ and clinical education (Section 7).^9^ Interpretation of this language implies that no training period would be categorized as work experience upon graduation.

On the other hand, many legal definitions of work experience are based upon whether there is financial compensation.^10^ Residents have employment contracts and job descriptions with assigned responsibilities and duties. Furthermore, they are postgraduates (many postdoctoral) with graduate diplomas. Thus, residents may be likened to postdoctoral researchers, who are largely accepted as workers rather than trainees. With this interpretation, an applicant could have 12 months (up to 24 months for a 3‐year residency) categorized as work experience upon successful completion of residency.

Between these two opposing viewpoints, there is a third interpretation regarding the duration of T&E required after residency. The stated CAMPEP methodology is to progressively increase resident responsibilities to the level of a practicing qualified medical physicist (Section 2.14).^9^ Even operating under direct supervision, this level of responsibility is largely indistinguishable from work experience. As a result, rather than the previous all (12 months)‐or nothing vantage points, this hybrid approach generates a range of T&E which can be counted toward NRC T&E requirements. In the standard 2‐year residency template, CAMPEP allocates 3 months to the final rotation where the resident is expected to function independently.^9^, ^11^ Up to 3 months could be categorized as work experience upon successful graduation, depending on factors such as pace, overall length of residency, and patient caseload.

## 3 USE OF TEMPLATE FORMS 313A(AMP) AND 313A(RSO/ARSO)

NRC Form 313A(AMP)^12^ and Form 313A(RSO/ARSO)^13^ each serve as a modular template application intended to provide a structured summary of an applicant's credentials relevant to AMP^3^ and RSO^4^ status, respectively. The standardization can streamline the review process for both the applicant and the reviewer. Visual block diagrams of these regulations and forms are shown in Figures 1 and 2.

With the T&E route, the applicant must complete multiple tables  with details on each subcategory of T&E such as the date and location of T&E. The attestation by a preceptor only considers if the applicant is able to independently fulfill the radiation safety‐related duties—there is no assessment of clinical proficiency included.^3^, ^4^, ^12^, ^13^ In addition to these T&E tables, Part II of each Form 313A requires attestation of the documented T&E.^12^, ^13^ The RSO T&E requires experience but this can include full‐time training and/or supervised work.^3^, ^4^ On the other hand, the AMP applicant must consider whether to consider time as either training or work experience^3^ (as discussed in Section 2 of this article and shown in Figures 1 and 2).

For NSB diplomates, T&E documentation in either Form 313A(AMP) and Form 313A(RSO/ARSO) is pared down to a single table^12^, ^13^ documenting the training dates and trainer for the specific use (i.e. RAU brachytherapy, Sr‐90, Teletherapy, and Gamma stereotactic radiosurgery) and the board certificate stamped with “AMP eligible” or “RSO/ARSO eligible.” Only a single table needs to be completed within the remaining balance of Form 313A (both part I and part II).^12^, ^13^

## 4 BASICS OF LIABILITY AND INDEMNITY

With the ABR serving as an NSB, institutions could simply delay their review period for an applicant until board certification, to avoid attestations entirely.^6^ Prior experience has demonstrated that attestations are practically inconvenient, time‐consuming, and potentially contentious for applicants. An NSB provides an objective alternate pathway for AMP and/or RSO eligibility which does not involve any attestations.^6^ Indeed, preceptors have expressed concern that they may be held responsible for the actions of those for whom they are attesting. While this can be construed as an intense focus on patient safety, it also points to some ambiguity in terms of liability for future actions of trainees. In general, any actions taken by a physicist after completion of training are considered to be independent of training (or attestations of their training).^14^ Physicists employed by an entity (i.e., hospital, clinic, or group practice), are commonly shielded through vicarious liability by the master insurance policy of their employer.^14^

While attestations are required in the regulations, these do not include any mention of clinical proficiency.^3^, ^4^ Rather, the attestations are solely limited to whether the applicant is able to independently fulfill the radiation safety‐related duties. Arguably, the definition of radiation safety‐related duties is loosely based on the complexity of procedures (for AMP) and/or procedural size and scope (for RSO).

Since this article is not meant to be a universal legal reference document, it is recommended to consult a legal professional for an individualized assessment. While an attestation ideally should be independent of future liability concerns, it may be useful to have more clarity regarding such liability in the NRC attestation forms.

## 5 CONSIDERATION OF OTHER AUTHORIZED PERSONNEL: PHYSICIAN AUTHORIZED USERS

Our physician counterparts can apply to become Authorized Users (AUs) and are evaluated by the NRC or NRC Agreement State based upon § 10 CFR Part 35.690,^15^ which requires physicians—regardless of board certification status—in the United States to have completed 3 years of a residency program which has been approved by the Residency Review Committee for Radiation Oncology of the Accreditation Council for Graduate Medical Education (ACGME).^16^ Upon finishing 3 years, physicians who have experience in radiation safety are also eligible to become an RSO if they already are on a license^4^ or can apply to the NRC or State simultaneously for both AU and RSO roles.^4^ Generally, the overlap of the AU and RSO functions, while legally supported, occurs mainly in low‐volume clinics.^17^

In regards to residencies, AU recognition is distinctly different from AMP/RSO because an accredited residency is required for physicians—even for those with board certification.^3^, ^4^, ^15^ Whereas, neither AMP nor RSO eligibility explicitly requires a residency as long as T&E is completed.^3^, ^4^ In fact, until 2014, residencies were not required for ABR physics board eligibility and there remain many current practicing AMP and/or RSOs who did not complete formal residency training.

The framework for AU effectively delegates the definition of proper T&E away from the NRC to the ACGME.^15^, ^16^, ^18^ The ACGME Radiation Oncology Program Requirements (PRs) for residents classify brachytherapy among its “core” competencies.^16^ ACGME T&E requirements are case‐based rather than time‐based. For example, for brachytherapy the ACGME requires at least seven interstitial (IS) and at least 15 intracavitary (IC) brachytherapy procedures.^16^ Of these 15 IC cases, a minimum of five must be tandem‐based insertions among at least two patients and, specifically, no more than 5 single‐channel vaginal cylinder cases will count toward the total 15 IC case requirement.^16^ The ACGME does not distinguish between training and work experience and, in fact, recognizes these experiences are generally gained concurrently.^15^, ^16^

## 6 POTENTIAL APPROACHES

The CAMPEP Residency Standards are publicly available documents which contain information on the minimum requirements of an accredited residency program.^9^ Section 8.9 of the latest version (released July 2024) contains a comprehensive list of topics to be covered.^9^ While some topics are discussed in TG249 (“Essentials and Guidelines for Clinical Medical Physics Residency Training Programs”),^7^ the topic outline from the CAMPEP Residency Standards does not include all AMP medical uses and is limited to RAU brachytherapy use.^8^, ^9^

There are no mandated details about the duration or depth of rotations except when other accrediting agencies dictate a minimum case‐load. However, the exception for accrediting agencies is not applicable for AMP or RSO T&E because neither the NRC nor the State are accrediting agencies. Encouragingly, CAMPEP requirements could be considered to satisfy the accrediting agency portion of this clause, but would likely require modification to be case‐load‐based. Of course, this would require the community to agree on an acceptable and objective minimum threshold for case‐load.

Some have suggested that portions of the AU paradigm be assimilated for physicists. One way would be if the NRC (or State) were to empower an accrediting body (i.e., CAMPEP) to periodically review and oversee the physics T&E. In this case, the language could be an adaption from NRC regulations^15^: “Successfully complete a minimum of 2 years of residency in a medical physics residency program approved by CAMPEP.” Alternatively, the NRC (or State) could change the time‐based clinical/work requirement to a case‐based requirement.

The use of case‐load as a metric for minimum training standards would indeed provide clarity and make a determination of whether an applicant has met the NRC eligibility requirements objective. However, this requires the unenviable task for an accrediting body to establish a consensus caseload: How many cases are adequate for a physicist to practice safely? How many cases are needed to establish confidence and competence?

## 7 CONCLUSIONS

The purpose of this article is not to provide a stance on interpretations, but rather to underscore the need for discussion and adoption of a uniform approach to eligibility of new residents for AMP or RSO status. While the T&E for RSO is straightforward, there has been a long‐standing difference in opinion among program directors and employers regarding the distinction between training and work for an AMP.^3^, ^4^, ^8^

One of the main advantages of board certification was an ironclad route to circumvent the T&E regulatory language and avoid attestations altogether. Consequently, the ABR decision to relinquish its NSB status has spurred a renewed, healthy dialogue which was not limited to T&E for AMP alone. A few of the emerging common topics shared among AMP and RSO were template forms, liability, and the parallel process for AUs. Indeed, the existing process for AU may provide an alternative wherein the NRC would empower CAMPEP to review and oversee the physics T&E.

There have been ongoing efforts to engage the medical physics community in discussing the implications of the ABR decision to withdraw from its NSB status. In August 2024, the NRC released a draft of a guidance document^19^ during the transition period from the NSB route to exclusively the T&E route. Unfortunately, this document did not provide clarification on the issues discussed in this article.

In addition to the NRC, there are two major professional societies who are aiming to raise awareness and foster a healthy dialogue: the Society of Directors of Academic Medical Physics Programs (SDAMPP) and the American Brachytherapy Society (ABS). To date, SDAMPP has already hosted three separate webinars which each outlined the issues included in this article.^20^, ^21^, ^22^ SDAMPP was specifically selected as its audience includes residency program directors and associate program directors, who are traditionally considered to be the most common attestors.^7^

In conjunction with SDAMPP educational efforts, the ABS has approved and convened a subcommittee to provide consensus definitions of AMP clinical brachytherapy experience and proficiency to satisfy NRC T&E for RAU brachytherapy use. This subcommittee was intentionally established through the ABS, which is considered the foremost authority for brachytherapy and hence has previously convened consensus groups dedicated to education and training.

There have been recent initiatives to raise awareness and foster healthy dialogue. Due to the remaining degree of existing ambiguity, we advocate for more awareness and continued dialogue amongst stakeholders to produce standardized definitions and provide clarity for the medical physics community.

## AUTHOR CONTRIBUTIONS

All authors participated and contributed to the data collection and this manuscript. Only those persons who contributed directly and significantly to the intellectual content of the paper are listed as authors. All authors contributed to the drafting, revision, and review of the content.

## CONFLICT OF INTEREST STATEMENT

The authors declare no conflicts of interest.

## APPENDIX A

### APPENDIX: Overall key “take‐away” points

- Prior to 2024, authorized medical physicist (AMP) and/or radiation safety officer (RSO) candidates could bypass documentation of Training and Experience (T&E) through board certification from a recognized Specialty Board, such as the American Board of Radiology (ABR) as shown in Figures 1 and 2. The withdrawal of the ABR as an NSB limits applicants boarded beginning in 2024 to the T&E route exclusively.
- AMP status requires 2 years of T&E, which is further subdivided into 1 year (12 months) of full‐time training, distinct from another year (12 months) of full‐time work experience.
- RSO T&E requires radiation safety “experience” (in addition to 200 hours of classroom and laboratory training in specific areas), but does not differentiate between training and work.
- While there is consensus that residencies fulfill the 1 year (12 months) full‐time training period, there is ambiguity about how much of the remaining residency period can be categorized as full‐time work experience, if any.
- Documentation of T&E via NRC Forms is neither consistent nor standardized for AMP and/or RSO status. Fortunately, attestation is limited only to an applicant's radiation safety capabilities. Legal protection through vicarious liability is afforded to many attestors.
- Authorized User (AU) status requires completion of 3 years of a residency program approved by a subcommittee of the Accreditation Council for Graduate Medical Education (ACGME) whose requirements are case‐load based. An analogous approach for AMP and RSO may be viable using the Commission on Accreditation of Medical Physics Education Programs (CAMPEP).
- Stakeholders such as the NRC, SDAMPP, and CAMPEP should be encouraged to convene or continue discussions and provide clarity. Other professional societies, such as the American Brachytherapy Society (ABS) have proactively convened a working group on AMP training.
